# Outcomes of transitioning from mandatory to elective research at a dental school in Southern California

**DOI:** 10.1002/jdd.13770

**Published:** 2024-11-12

**Authors:** So Ran Kwon, Udochukwu Oyoyo, Mark Estey

**Affiliations:** ^1^ Division of General Dentistry Loma Linda University School of Dentistry Loma Linda California USA; ^2^ Dental Educational Services Loma Linda University School of Dentistry Loma Linda California USA; ^3^ Academic Affairs Loma Linda University School of Dentistry Loma Linda California USA

**Keywords:** dental education, faculty, perception, research, students

## Abstract

**Objectives:**

The purpose of this study was to assess the impact of a curricular change from mandatory to elective research on the student research outcomes at a Southern California Dental School over ten years. Additionally, the perception of dental students and faculty toward research in dental education was assessed.

**Methods:**

A survey was distributed to determine the perception towards research in dental education, motivation and barriers for pursuing research and the possibility participants would pursue research—even if the school did not mandate it. The survey was distributed to a total of 507 dental students and 121 full‐time faculty.

**Results:**

Both dental students (80.6%) and faculty (91.4%) agreed that learning about research is important in dental education. Both cohorts generally agreed that research experience enhances dental training, supports the scientific basis of dental treatment methods, and correlates with clinical ability. The majority of students (77.0%) disagreed with mandating research while faculty (77.6%) believed that it should be mandated. Lack of time was the single most stated barrier for participating in research. The overall trend during the transition from mandatory to elective research showed that initially there was a stark drop in students and faculty participation and projects being carried out. However, with deliberate planning, research outcomes steadily increased.

**Conclusions:**

We conclude that dental students and faculty have a positive perspective on the importance of research in dental education and actively engage in research and mentoring activities when provided with a supportive and encouraging environment. This participation occurs regardless of whether research is mandated or elective.

## 1 INTRODUCTION

Scientific research is the foundation of evidence‐based dentistry, driving the education and practices that enable evidence‐based decision‐making.^1^ The Commission on Dental Accreditation (CODA) emphasizes the importance of research in Standard 6, which states: “Dental education programs must provide opportunities, encourage, and support student participation in research and other scholarly activities mentored by faculty.^2^” Consequently, most dental schools include some kind of research training in their curricula to ensure students understand the value of research, learn to interpret data and recognize that the future of the profession relies on ongoing research.^3^, ^4^, ^5^, ^6^

Loma Linda University School of Dentistry (LLUSD) actively promotes student research opportunities. In the four‐year curriculum, there are three courses—research design, biostatistics, and research laboratory—that are mandated for third‐year students during the summer and autumn quarters. These courses provide an overview of evidence‐based dentistry and teach basic concepts and principles related to evaluating and conducting research in dental education and biomedical sciences. Students work in a group of 3–4 students and are assigned to a mentor who guides them in the write‐up of the research proposal and conduct of the project over two quarters. Through the process, students recognize research problems, search and review relevant literature, interpret results, and draw proper conclusions based on the best evidence. Completing a research project is mandatory and also involves presenting locally at LLU Homecoming and at regional conferences in Southern California.

A significant change in the LLUSD curriculum occurred in 2015 when completing a research project transitioned from mandatory to elective. Students are still required to take the research design and biostatistics classes, but they no longer have to complete a science‐based experimental project unless they choose to take the elective research laboratory class. This change was based on several reasons: a shortage of available mentors for around 100 students (about 25 research teams) and student feedback indicating that, while learning the concepts is important, carrying out the project is too demanding given the limited time available for research.

With the transition from mandatory to elective research participation, this study was initiated to assess the impact of the curricular change on the student research outcomes over 10 years and assess the perception of LLUSD students and faculty toward research in dental education. We hypothesized that there would be no difference in the perception towards research in dental education among two LLUSD cohorts: dental students and faculty.

## 2 MATERIALS AND METHODS

The distribution of an anonymous survey was approved by the Institutional Review Board of Loma Linda University (#5160424) and the Office of Educational Assessment of LLUSD.

The survey consisted of 10 closed‐ended questions that included respondents’ demographics, perception towards research in dental education, motivation and barriers for pursuing research and the possibility participants would pursue research—even if the school did not mandate it. Responses for perception questions were on a 5‐point Likert scale with 1 = strongly disagree, 2 = disagree, 3 = neither agree nor disagree, 4 = agree, and 5 = strongly agree. The survey was distributed as a hard copy to a total of 507 dental students (DDS Class of 2019–2023) and 121 full‐time faculty over 5 years during the annual LLU Homecoming convention in 2017 through 2021. Each DDS class completed the survey during their 2nd year, as they were required to attend the Homecoming event to observe their upperclassmen present their research projects.

The 10‐year retrospective data on outcomes of student research, including the number of participating students, mentors, research projects, and publications, were provided by the student research office.

All data were entered into an Excel spreadsheet and descriptive statistics were compiled. A series of Chi‐square tests of independence were conducted to examine whether there were differences in the responses to perception towards research in dental education: Questions 1 through 5 between dental students and faculty. Statistical inferences were made based on a 5% significance level for all tests. Data were analyzed using Jamovi software 2.5.4.^7^

## 3 RESULTS

**FIGURE 1:**
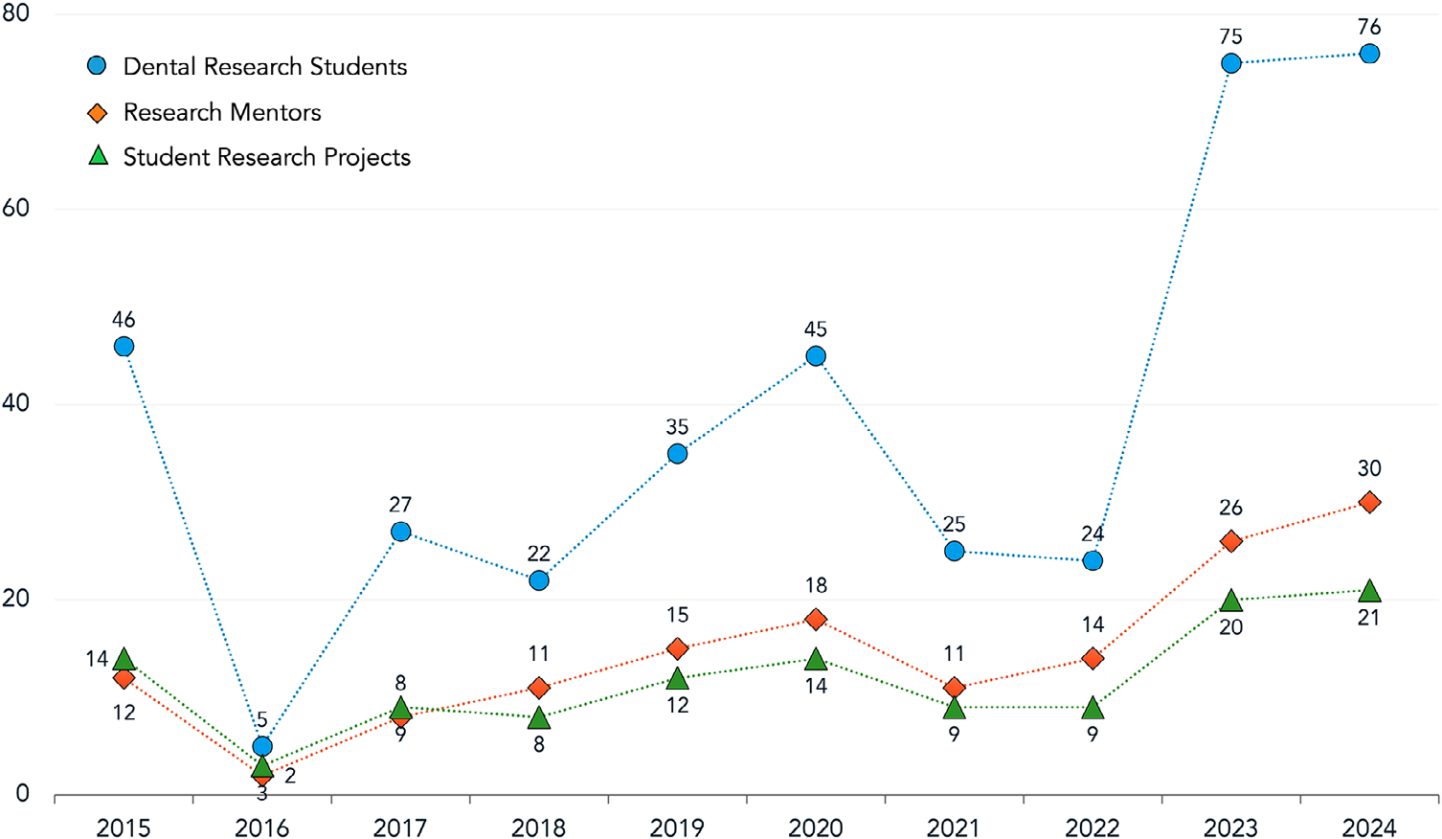
Summary of number of dental students and mentors participating in research, and projects over 10 years.

The 10‐year retrospective review of a number of dental students and mentors participating in research, and projects carried out by year is illustrated as line plots in Figure 1. Even though carrying out a research project was mandated in 2015, many students could not complete a project due to the shortage of faculty mentors. This prompted curricular changes to transition from mandatory to elective research. With this change, the number of dental research students experienced a sharp drop from 46 in 2015 to five in 2016, followed by a general upward trend, peaking at 76 in 2024. Similar trends were observed regarding the number of research mentors which showed a steady increase, starting at two in 2016 and rising to 30 by 2024, with a notable decline in 2021 which was attributed to the coronavirus disease 2019 pandemic. The number of dental student research projects fluctuated initially but demonstrated a steady upward trajectory from three in 2016 to 21 in 2024. These trends highlighted a growing involvement and support in dental student research activities despite curricular changes.

**FIGURE 2:**
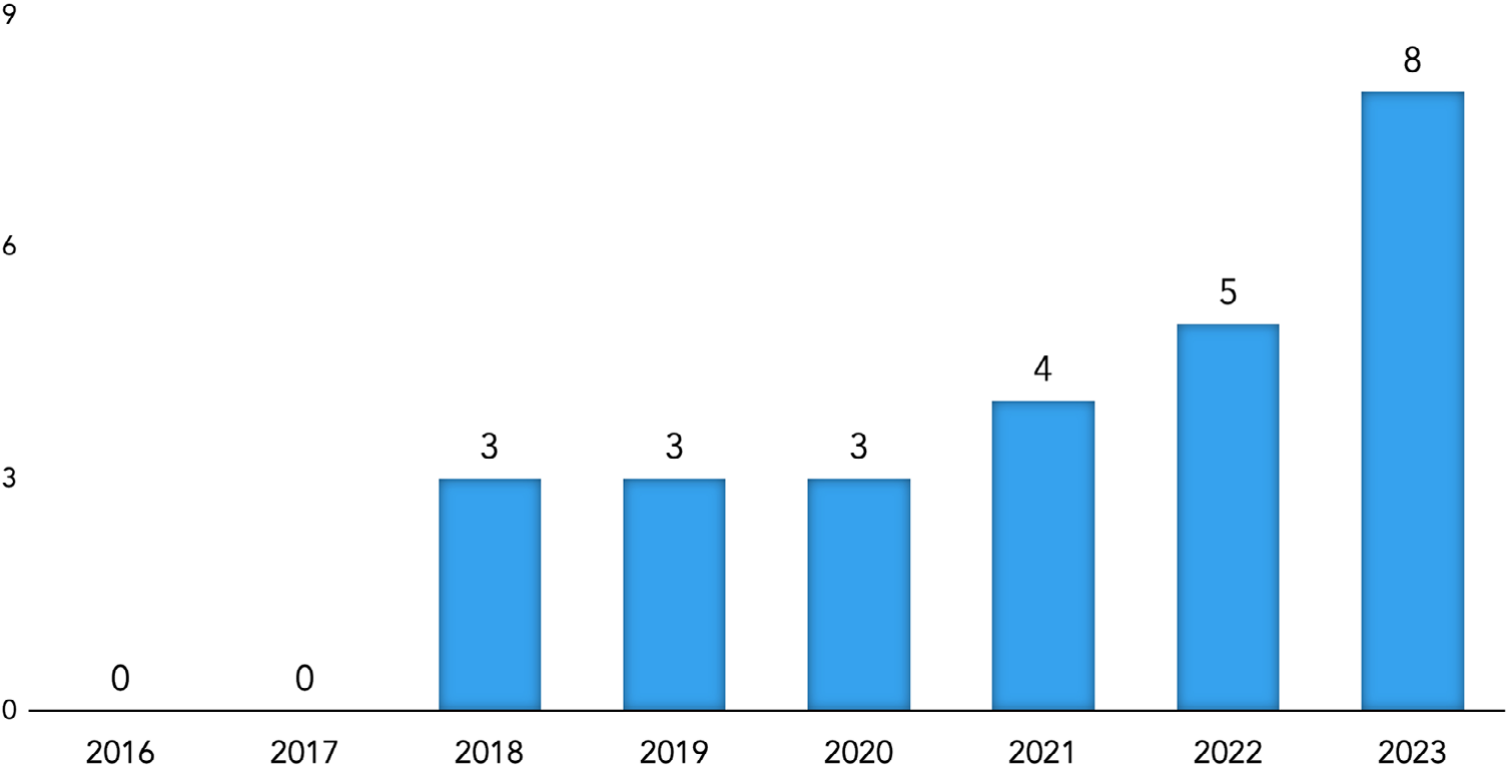
Number of student research publications by year.

The trend in student research publications from 2016 to 2023 is illustrated in Figure 2. There were no publications recorded in 2016 and 2017. From 2018 to 2020, the number of publications remained steady at three per year. A gradual increase was observed with four publications in 2021 and five in 2022. The most significant rise occurred in 2023, with the peak number of eight student research publications in peer‐reviewed journals.

**FIGURE 3:**
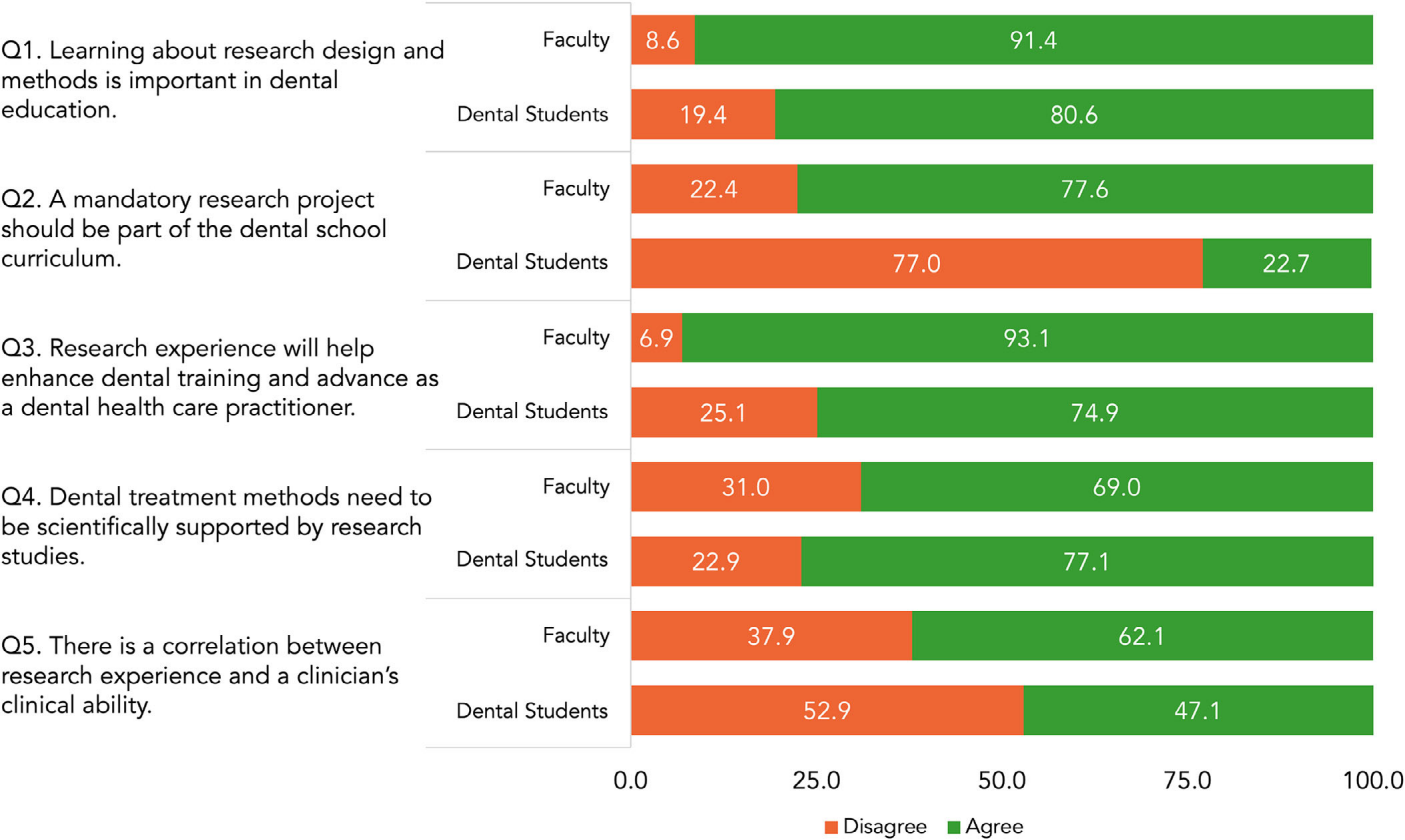
Perception towards research in dental education: faculty versus dental students.

The response rate for the survey was 96.4% for dental students and 47.9% for faculty. The bar graph in Figure 3. summarizes the responses of dental students (*N* = 489, Class of 2019–2023) and faculty (*N* = 58) to five questions regarding their perception towards research in dental education. Perception responses were on a 5‐point Likert scale ranging from strongly disagree to strongly agree. For the analysis, we dichotomized the responses into positive and negative, with the “neutral” response categorized into the negative response. Typically, in survey research, neutral responses can sometimes reflect indifference or a reluctance to choose a more definitive position (either positive or negative). For this reason, we opted to dichotomize neutral responses along with negative responses to provide a clearer contrast between those who showed definitive engagement with the research initiative (positive responses) and those who did not (neutral and negative responses).^8^

The Chi‐square test of independence showed that there was no significant association between the two cohorts on the importance of learning about research design and methods in dental education (Q1). *χ*2(1, N = 547) = 3.48, *p* = 0.062. The proportion of positive responses was 80.4% (393/489) for dental students and 91.4% (53/58) for faculty. There was a significant difference in responses between dental students and faculty for Q2 regarding the inclusion of a mandatory research project in the dental curriculum and the benefits of research experience in enhancing dental training (Q3). Specifically, faculty were more likely than dental students to support a mandatory research project (77.6% vs. 22.7%, *χ*2[1, *N* = 546] = 73.73, *p* < 0.001) and to view research experience as beneficial (93.1% vs. 75.7%, χ2[1, *N* = 547] = 8.07, *p* = 0.004). For questions about the necessity of scientific support for dental treatments (Q4), and the correlation between research experience and clinical ability (Q5), no significant differences were found between the cohorts (*p* > 0.05, in both instances).

The responses of dental students to motivators in pursuing research were diverse. The most common reason, chosen by 35% of respondents, was the belief that research would help them become life‐long learners. About 30% of respondents had no interest in research and 20% pursued research due to their interest in the field. Approximately 10% did research to improve their chances of getting into residency programs. Only a very small number of respondents (1.7%) felt that research was not relevant to dental school education.

The most common barrier to performing research among dental students was a lack of time (66.4%), followed by a lack of motivation (11.2%), lack of incentive (6.2%), and lack of application/use (5.8%). When asked if they would still pursue research even if it was not mandated by the school, approximately 22% of respondents indicated they would, 25% said they would not, and 53% said ‘Maybe’.

## 4 DISCUSSION

Dental schools have been proactive in reviewing and updating their curricula.^9^, ^10^ These curricular changes are needed to respond to scientific advancements, societal needs, regulatory and accreditation requirements, and stakeholder feedback. These changes should also actively engage students, emphasizing the roles, responsibilities, and common tasks of dental practitioners.^11^ The transition at LLUSD from mandatory to elective research participation was primarily due to a shortage of available faculty mentors. While the exact reasons for this shortage were not investigated, it is largely attributed to faculty burnout and the lack of providing additional time for mentoring duties.

The 10‐year trend of LLUSD student research showed that abrupt curricular changes without strategic planning can be grim, as evidenced in 2016 when only three student research projects were conducted across the entire dental school. Yet, despite the significant drop, there has been a gradual increase in dental student research activities, which may be attributed to the general belief among students and faculty that research in dental education is important for advancing the field and preparing students for lifelong learning.^12^ However, it was clear that dental students were less likely than faculty to support mandatory research and to see research experience as beneficial, leading us to reject our hypothesis. Furthermore, only 22% of dental students indicated they would undertake a research project if it was optional. This was in contrast with another US dental school in the East, where 48% of students said they would definitely pursue research even if it was not compulsory.^13^ Interestingly, despite many dental students initially being undecided or opposed to engaging in research, the overall trend at LLUSD shifted significantly. This is reflected by the gradual increase in dental students participating in research with a peak of 76 students participating in research by 2024.

It is challenging to pinpoint specific reasons for this commendable growth. However, a change in the leadership of the student research program in 2016 included deliberate planning to enhance research participation among students and faculty and to create a supportive and collaborative research environment. Despite students noting a ‘lack of time’ as the largest barrier to performing research, consistent with findings from other dental schools,^12^, ^14^ discussions with the LLUSD clinic and academic administration did not lead to a resolution. The tightly packed curriculum did not allow sufficient space for incorporating research experiences.

Therefore, creative solutions were necessary, with the primary goal being to instill enthusiasm in dental students, as this is considered crucial for developing new researchers.^15^ To motivate and create enthusiasm for research participation, the first step was recognizing the importance of catering to unique interest areas. Each student has specific interests in research topics and study designs, making it vital to offer a broad range of clinically relevant research topics. Second, providing diverse mentorship opportunities was addressed by compiling a list of faculty mentors from various departments covering scientific, clinical, and community‐based studies before the start of the research design class. This allowed students to collaborate with mentors matching their interests. Third, promoting core values of excellence and teamwork was achieved by encouraging students to work in groups with their peers, synergizing their efforts to collaboratively build excellence. Finally, encouraging presentation and publication involved having students present their work at Homecoming and regional dental conferences to share their research efforts but also allow them to interact with peers and oral health care professionals. Several sessions on writing and publishing in peer‐reviewed journals were offered to create a supportive environment. Specifically, the Journal of the California Dental Association dedicates an issue to research articles by dental students and their faculty mentors each year.^16^ Students were highly encouraged to take advantage of this opportunity.

Reflecting on the student research outcomes, highlighted the critical role of mentors in guiding students through their research projects, emphasizing the need to reignite a research culture and enthusiasm among the faculty. To achieve this, several strategies were implemented. First, diverse faculty members were targeted, including clinical faculty from various departments, rather than relying solely on research faculty. Second, the responsibilities and benefits of mentoring were proactively communicated through presentations at faculty and departmental meetings. Third, recognizing student research mentorship as an important scholarly activity for promotion was accomplished by collaborating with the promotions committee, although no built‐in mentoring time was allotted for this. Fourth, recognition and appreciation initiatives such as ‘Student Research Mentors Appreciation Day’ and ‘Student Research Mentor of the Year’ were established to formally acknowledge faculty contributions. Finally, a faculty development program was established to train and guide junior faculty in mentoring research projects.^17^ These collective efforts aimed to create an uplifting research culture, and thereby engage dental students and faculty for lifelong learning and advancing the future of the dental profession.

## 5 CONCLUSIONS

To maintain the vitality of student research participation, future directions should emphasize strategic planning and curricular adjustments that integrate research activities without overburdening students or faculty. We conclude that dental students and faculty hold a positive perspective on the importance of research in dental education and actively engage in research and mentoring activities when provided with a supportive and encouraging environment. This participation occurs regardless of whether research is mandated or elective.
